# Ventilator-induced endothelial activation and inflammation in the lung and distal organs

**DOI:** 10.1186/cc8168

**Published:** 2009-11-16

**Authors:** Maria A Hegeman, Marije P Hennus, Cobi J Heijnen, Patricia AC Specht, Burkhard Lachmann, Nicolaas JG Jansen, Adrianus J van Vught, Pieter M Cobelens

**Affiliations:** 1 Laboratory of Psychoneuroimmunology, University Medical Center Utrecht, Lundlaan 6, Utrecht, 3584 EA, the Netherlands; 2 Department of Pediatric Intensive Care, University Medical Center Utrecht, Lundlaan 6, Utrecht, 3584 EA, the Netherlands; 3 Department of Anesthesiology, Erasmus Medical Center, Dr. Molewaterplein 50-60, Rotterdam, 3015 GE, the Netherlands; 4 Department of Anesthesiology and Intensive Care Medicine, Charité Campus Virchow-Klinikum, Humboldt-University, Augustenburger Platz 1, Berlin, D-13353, Germany (current address); 5 Department of Intensive Care Medicine, University Medical Center Utrecht, Heidelberglaan 100, Utrecht, 3584 CX, the Netherlands

## Abstract

**Introduction:**

Results from clinical studies have provided evidence for the importance of leukocyte-endothelial interactions in the pathogenesis of pulmonary diseases such as acute lung injury (ALI) and acute respiratory distress syndrome (ARDS), as well as in systemic events like sepsis and multiple organ failure (MOF). The present study was designed to investigate whether alveolar stretch due to mechanical ventilation (MV) may evoke endothelial activation and inflammation in healthy mice, not only in the lung but also in organs distal to the lung.

**Methods:**

Healthy male C3H/HeN mice were anesthetized, tracheotomized and mechanically ventilated for either 1, 2 or 4 hours. To study the effects of alveolar stretch *in vivo*, we applied a MV strategy that causes overstretch of pulmonary tissue i.e. 20 cmH_2_O peak inspiratory pressure (PIP) and 0 cmH_2_0 positive end expiratory pressure (PEEP). Non-ventilated, sham-operated animals served as a reference group (non-ventilated controls, NVC).

**Results:**

Alveolar stretch imposed by MV did not only induce *de novo *synthesis of adhesion molecules in the lung but also in organs distal to the lung, like liver and kidney. No activation was observed in the brain. In addition, we demonstrated elevated cytokine and chemokine expression in pulmonary, hepatic and renal tissue after MV which was accompanied by enhanced recruitment of granulocytes to these organs.

**Conclusions:**

Our data implicate that MV causes endothelial activation and inflammation in mice without pre-existing pulmonary injury, both in the lung and distal organs.

## Introduction

Critically ill patients in the intensive care unit often require mechanical ventilation (MV) to adequately oxygenate vital organs. Although artificial ventilation is lifesaving, the procedure itself may lead to serious damage in both healthy and diseased lungs [1]. Studies have revealed that the cyclic opening and collapse of alveoli during MV may provoke alveolar stretch and subsequently result in ventilator-induced lung injury (VILI) [2,3]. Important features of VILI are increased cytokine or chemokine production, alveolar-capillary permeability, protein-rich edema formation and, ultimately, impaired gas exchange [4-6].

Pro-inflammatory cytokines such as IL-1β and TNF-α are secreted by alveolar macrophages upon mechanical stretch [7] and are capable of stimulating endothelial activation [8]. In turn, cytokine-activated endothelial cells secrete chemokines and express adhesion molecules on their surface resulting in enhanced leukocyte adhesiveness and transmigration of activated immune cells across the endothelium of inflamed tissue [8-10]. Vascular adhesion molecules that belong to the selectin family (P-selectin and E-selectin) mediate leukocyte margination and rolling along the blood vessel wall, whereas members of the immunoglobulin (Ig) superfamily (vascular cell adhesion molecule (VCAM)-1, intercellular adhesion molecule (ICAM)-1 and platelet-endothelial cell adhesion molecule (PECAM)-1) participate in leukocyte adhesion and transmigration into underlying tissue [11]. Previously, it has been shown that soluble adhesion molecule levels are elevated in patients with serious lung diseases such as acute lung injury (ALI) and acute respiratory distress syndrome (ARDS) [12,13]. Moreover, augmented P-selectin, VCAM-1 and ICAM-1 expression was found in pulmonary tissue after MV [14] suggesting that adhesion molecules may play a crucial role in the pathogenesis of VILI.

Most critically ill patients do not succumb to lung deterioration associated with MV but to multiple-organ failure (MOF) caused by a systemic inflammatory response syndrome [15,16]. As a mechanism of MOF, it has been hypothesized that ventilator-induced lung inflammation may elicit release of inflammatory mediators into the circulation, thereby amplifying a pro-inflammatory systemic environment and eventually leading to detrimental effects in distal organs [17-19]. As high levels of inflammatory mediators in the periphery are believed to be important in the pathogenesis of MOF [20,21], systemic effects of MV have been proposed to be responsible [18,19]. Similar to sepsis-induced MOF [22,23] activation of endothelial cells in distal organs might be essential in the development of ventilator-induced MOF.

We designed this study to investigate whether ventilator-induced alveolar stretch may cause endothelial activation in healthy mice, not only in the lung but also in organs distal to the lung. To determine endothelial activation in these organs we assessed *de novo *synthesis of adhesion molecules. Moreover, we examined ventilator-induced effects on the inflammatory state of pulmonary, hepatic, renal and cerebral tissue.

## Materials and methods

### Animals

The experiments were performed in accordance with international guidelines and approved by the experimental animal committee of the Erasmus Medical Center Rotterdam. A total of 42 adult male C3H/HeN mice (Harlan CPB, Zeist, the Netherlands), weighing 25 to 30 g, were randomly assigned to different experimental groups.

To investigate the effects of alveolar stretch *in vivo*, we applied a MV strategy that has been described to cause overstretch of pulmonary tissue [24,25]. The method of MV was based on the first experiments performed in mice [26]. Thirty mice were tracheotomized under inhalation anesthesia (65% nitrous oxide, 33% oxygen, 2% isoflurane; Pharmachemie, Haarlem, the Netherlands). Subsequently, anesthesia was continued with 24 mg/kg/h intraperitoneal sodium pentobarbital (Algin, Maassluis, the Netherlands). Additional anesthesia was given when necessary. The intraperitoneal administered anesthesia fluid was sufficient to correct for hypovolemia. Muscle relaxation was attained with 0.4 mg/kg/h intramuscular pancuronium bromide (Organon Technika, Boxtel, the Netherlands). The animals were connected to a Servo Ventilator 300 (Siemens-Elema, Solna, Sweden) and ventilated for one, two or four hours in a pressure-controlled time-cycled mode (n = 9 to 10 per group), at a fractional inspired oxygen concentration (FiO_2_) of 1.0, inspiration to expiration ratio of 1:2 and frequency of 20 to 30 breaths/min to maintain normocapnia. Peak inspiratory pressure (PIP) was set at 20 cmH_2_O and positive end-expiratory pressure (PEEP) at 0 cmH_2_O. A polyethylene catheter was inserted into the carotid artery and blood gas determinations were performed using a pH/blood gas analyzer (ABL 505; Radiometer, Copenhagen, Denmark). Body temperature was maintained between 36 and 38°C with a heating device (UNO Roestvaststaal, Zevenaar, the Netherlands). Eight healthy non-ventilated, sham-operated mice served as a reference group (non-ventilated controls (NVC)). To investigate whether the high partial pressure of arterial oxygen (PaO_2_) levels associated with our MV strategy may contribute to changes in the immune response, spontaneously breathing animals (n = 6) were placed in an oxygen saturated box for four hours (FiO_2 _of 1.0, hyperoxia). This exposure time was chosen, because it resembles the longest period of time that mice were subjected to MV. All animals were sacrificed with an overdose of intraperitoneal sodium pentobarbital (Organon, Oss, the Netherlands).

### Histology

Two mice per group were perfused with PBS. Pulmonary tissue was directly removed, frozen in liquid nitrogen and stored at -80°C to evaluate lung architecture and presence of granulocytes. Before being snap frozen, lungs were filled with Tissue-Tek (Sakura Finetek, Zoeterwoude, the Netherlands).

Cryosections (5 μm) were cut on a cryostat microtome (Leica Microsystems, Nussloch, Germany) and fixed with acetone for 10 minutes. To assess pulmonary histopathology, longitudinal sections were stained with H&E (Klinipath, Duiven, the Netherlands).

### Tissue homogenates

Pulmonary, hepatic, renal and cerebral tissue (from four to eight mice per group) was directly removed and frozen in liquid nitrogen to evaluate endothelial activation and inflammation. Tissues were pulverized using a liquid nitrogen-cooled mortar and pestle, divided in several fractions and stored at -80°C allowing us to use the lung, liver, kidney and brain from one animal for multiple analyses. All analyses were performed in a blinded-setup.

### Myeloperoxidase assay

For lung and brain, myeloperoxidase (MPO) activity was determined as described previously [27]. In short, pulverized tissues were homogenized in 50 mM HEPES buffer (pH 8.0), centrifuged and pellets were homogenized again in water and 0.5% cetyltrimethylammonium chloride (Merck, Darmstadt, Germany). After centrifugation, supernatants were diluted in 10 mM citrate buffer (pH 5.0) and 0.22% cetyltrimethylammonium chloride. A substrate solution containing 3 mM 3',5,5'-tetramethylbenzidine dihydrochloride (Sigma-Aldrich, Steinheim, Germany), 120 μM resorcinol (Merck, Darmstadt, Germany) and 2.2 mM hydrogen peroxide (H_2_O_2_) in distilled water was added. Reaction mixtures were incubated for 20 minutes at room temperature and stopped by addition of 4 M sulfuric acid (H_2_SO_4_) followed by determination of optical density at 450 nm. MPO activity of a known amount of MPO units (Sigma-Aldrich, Steinheim, Germany) was used as reference. For liver and kidney, pulverized tissues were homogenized in lysis buffer with protease inhibitors. In supernatants MPO activity was analyzed by ELISA according to the manufacturer's instructions (Hycult Biotechnology, Uden, the Netherlands). To correct for homogenization procedures, total protein concentration of samples was determined with a BCA protein-assay (Pierce Biotechnology, Rockford, IL, USA) using BSA as standard.

### Quantitative real-time RT-PCR analysis

Total RNA was isolated from pulverized tissues with TRIzol^® ^reagent (Invitrogen, Paisley, UK). cDNA was synthesized from total RNA with SuperScript Reverse Transcriptase kit (Invitrogen, Paisley, UK). Quantitative real-time RT-PCR reaction was performed with iQ5 Real-Time PCR Detection System (Biorad, Hercules, CA, USA) using primers for E-selectin, VCAM-1, ICAM-1, PECAM-1, IL-1β, TNF-α and keratinocyte-derived chemokine (KC; murine homologue of IL-8; Table 1). To confirm appropriate amplification, size of PCR products was verified by agarose gel separation. Data were normalized for expression of internal controls β-actin and glyceraldehyde 3-phosphate dehydrogenase.

**Table 1:** Primers used for quantitative real-time RT-PCR

	Forward	Reverse
E-selectin	CAACgTCTAggTTCAAAACAATCAg	TTAAgCAggCAAgAggAACCA
VCAM-1	TgAAgTTggCTCACAATTAAgAAgTT	TgCgCAgTAgAgTgCAAggA
ICAM-1	ggAgACgCAgAggACCTTAACAg	CgACgCCgCTCAgAAgAACC
PECAM-1	ACgATgCgATggTgTATAAC	ACCTTgggCTTggATACg
IL-1β	CAACCAACAAgTgATATTCTCCATg	gATCCACACTCTCCAgCTgCA
TNF-α	gCggTgCCTATgTCTCAg	gCCATTTgggAACTTCTCATC
KC	AAAAggTgTCCCCAAgTAACg	gTCAgAAgCCAgCgTTCAC
β-actin	AgAgggAAATCgTgCgTgAC	CAATAgTgATgACCTggCCgT
GAPDH	TgAAgCAggCATCTgAggg	CgAAggTggAAgAgTgggAg

GAPDH = glyceraldehyde 3-phosphate dehydrogenase; ICAM = intercellular adhesion molecule; IL = interleukin; KC = keratinocyte-derived chemokine; PECAM = platelet-endothelial cell adhesion molecule; TNF = tumor necrosis factor; VCAM = vascular cell adhesion molecule.

### Statistical analysis

Data are expressed as mean ± standard error of the mean. All parameters were analyzed by one-way analysis of variance (ANOVA) with Least Significant Difference (LSD) post-test. *P*-values less than 0.05 were considered statistically significant.

## Results

### Stability of the model

**Figure 1:**
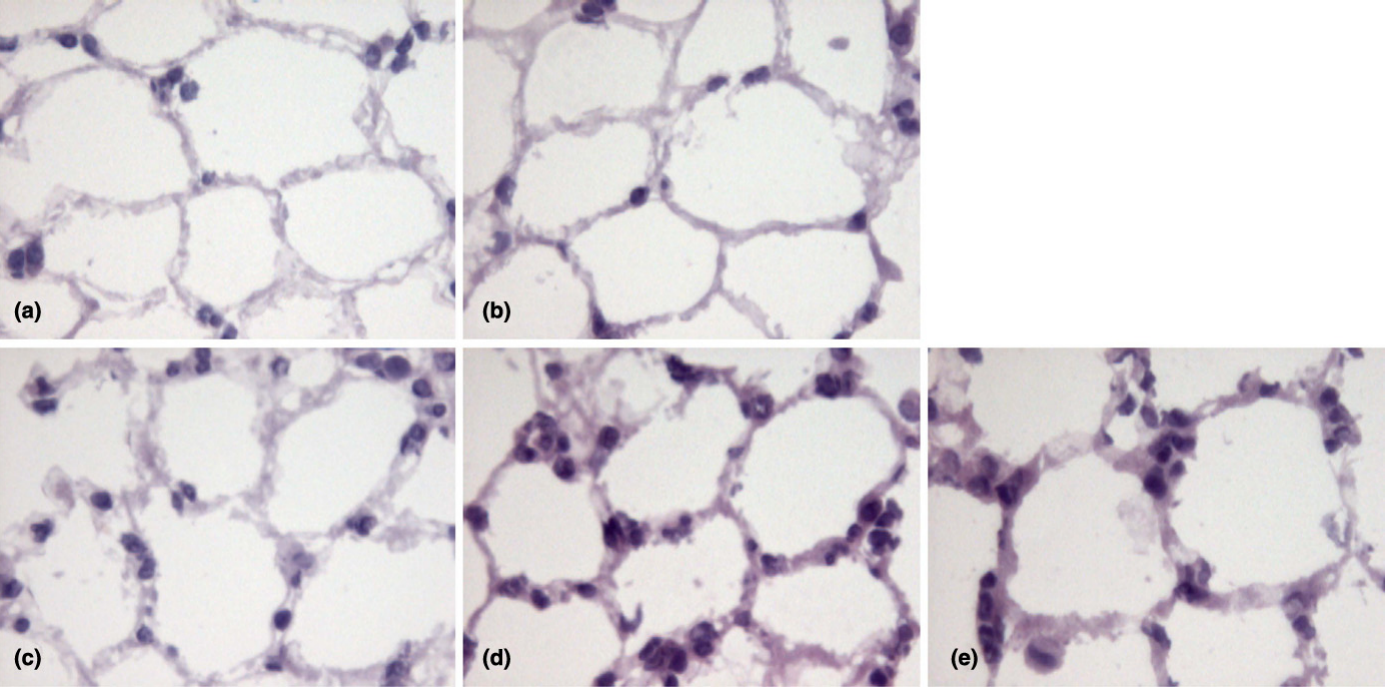
Histopathology of pulmonary tissue. Frozen lung sections were stained with H&E to analyze lung architecture and presence of granulocytes in pulmonary tissue. (a) Non-ventilated controls, (b) mice exposed to hyperoxia for four hours, and mice mechanically ventilated for (c) one, (d) two and (e) four hours. Magnification ×500.

MV was applied to healthy mice to induce alveolar stretch. All mice survived the ventilatory protocol and produced urine throughout the experiment. Arterial blood gas analysis of ventilated mice showed a stable oxygen tension (PaO_2_) with carbon dioxide tension (PaCO_2_), pH and base excess (BE) within the physiological range (Table 2). In addition, pulmonary architecture was preserved during the experiment (Figure 1).

**Table 2:** Oxygenation variables after one, two and four hours of mechanical ventilation

Time (hours)	PaO_2 _(mmHg)	PaCO_2 _(mmHg)	pH	BE
1	568.6 ± 23.1	31.0 ± 2.8	7.46 ± 0.02	-0.8 ± 1.4
2	509.7 ± 22.0	35.4 ± 2.3	7.42 ± 0.02	-1.5 ± 0.6
4	493.4 ± 24.9	45.4 ± 4.3	7.32 ± 0.03	-4.0 ± 0.9

Data are presented as mean ± standard error of the mean. BE = base excess; PaCO_2 _= partial pressure of arterial carbon dioxide; PaO_2 _= partial pressure of arterial oxygen.

### Effects of MV on inflammatory state of pulmonary tissue

#### Endothelial activation

**Figure 2:**
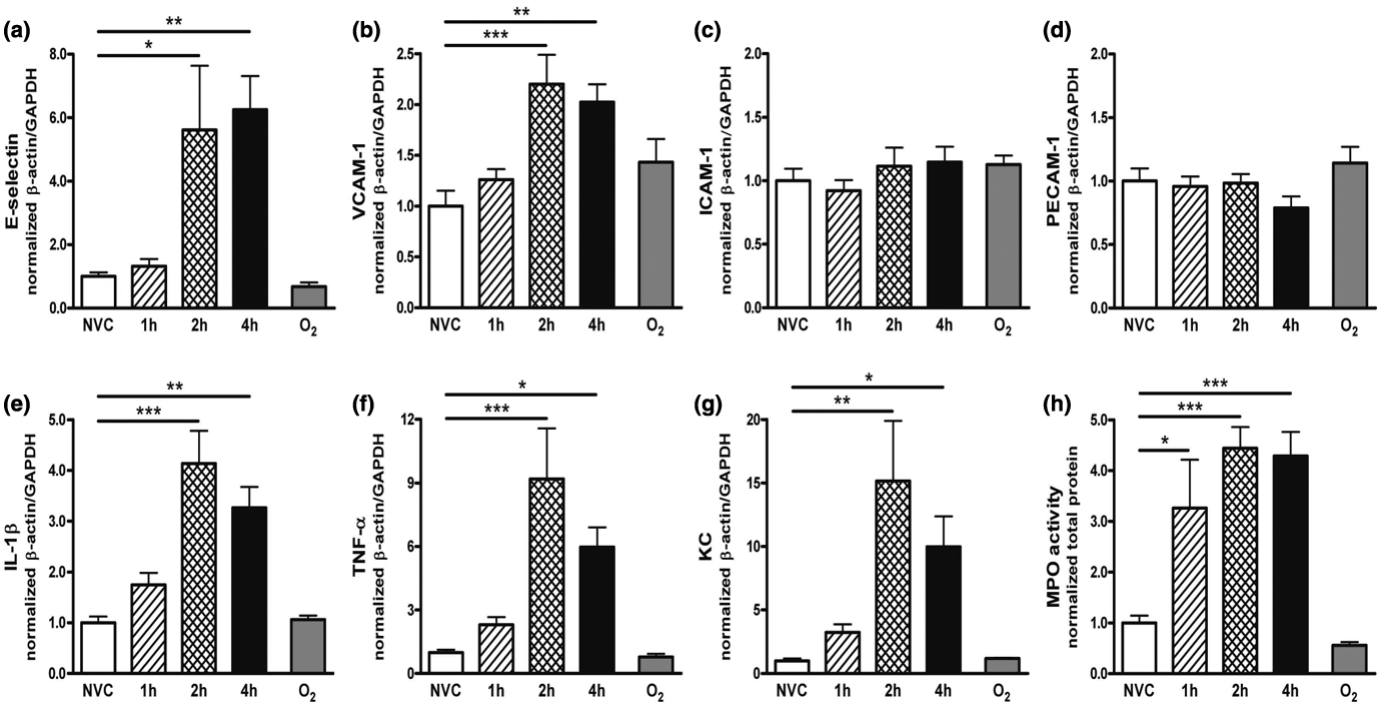
Ventilator-induced endothelial activation and inflammation in pulmonary tissue. In total lung homogenates, mRNA expression of the adhesion molecules (a) E-selectin, (b) vascular cell adhesion molecule (VCAM)-1, (c) intercellular adhesion molecule (ICAM)-1 and (d) platelet-endothelial cell adhesion molecule (PECAM)-1 was determined by quantitative real time RT-PCR. In addition, we studied ventilator-induced pulmonary inflammation by measuring mRNA expression of the pro-inflammatory cytokines (e) IL-1β and (f) TNF-α and the chemokine (g) keratinocyte-derived chemokine (KC). In total lung homogenates, (h) myeloperoxidase (MPO) activity was determined as a measure of granulocyte infiltration. Data are expressed as mean ± standard error of the mean of four to eight mice for each group (* *P *< 0.05, ** *P *< 0.01, *** *P *< 0.001 vs. non-ventilated controls (NVC)). 1 h = mechanically ventilated for one hour; 2 h = mechanically ventilated for two hours; 4 h = mechanically ventilated for four hours; O_2 _= hyperoxia for four hours; GAPDH = glyceraldehyde 3-phosphate dehydrogenase.

We studied the effect of MV on endothelial activation in pulmonary tissue by measuring *de novo *synthesis of adhesion molecules. Compared with NVC, enhanced mRNA expression of E-selectin and VCAM-1 was noticed after two and four hours of MV (Figures 2a and 2b). No ventilator-induced changes in ICAM-1 and PECAM-1 mRNA were found in the lung (Figures 2c and 2d).

#### Cytokine and chemokine expression

After two and four hours of MV, significantly higher mRNA expression of the pro-inflammatory cytokines IL-1β and TNF-α were observed (Figures 2e and 2f). In addition, MV induced an increase in mRNA expression of the chemokine KC (Figure 2g).

#### Granulocyte recruitment

To investigate whether the ventilator-induced endothelial activation and chemokine expression was accompanied by recruitment of granulocytes to inflamed pulmonary tissue, MPO activity was determined in total lung homogenates (Figure 2h). Elevated MPO activity was found after one, two and four hours of MV, which correlated with the presence of granulocytes observed in frozen pulmonary sections stained for H&E (Figure 1). Furthermore, histology of ventilated lungs showed margination of granulocytes to the blood vessel wall. Exudation of granulocytes into the alveolar space was not observed.

#### Contribution of hyperoxia

To examine whether the high PaO_2 _levels associated with our MV strategy contributed to changes in the pulmonary immune response, we exposed spontaneously breathing mice to 100% oxygen levels for four hours (FiO_2 _of 1.0, hyperoxia). As depicted in Figures 1 and 2, hyperoxia-exposed mice (O_2 _group) showed a similar adhesion molecule, cytokine and chemokine expression, MPO activity and lung histopathology compared with NVC.

### Effects of MV on inflammatory state of hepatic, renal and cerebral tissue

#### Endothelial activation

**Figure 3:**
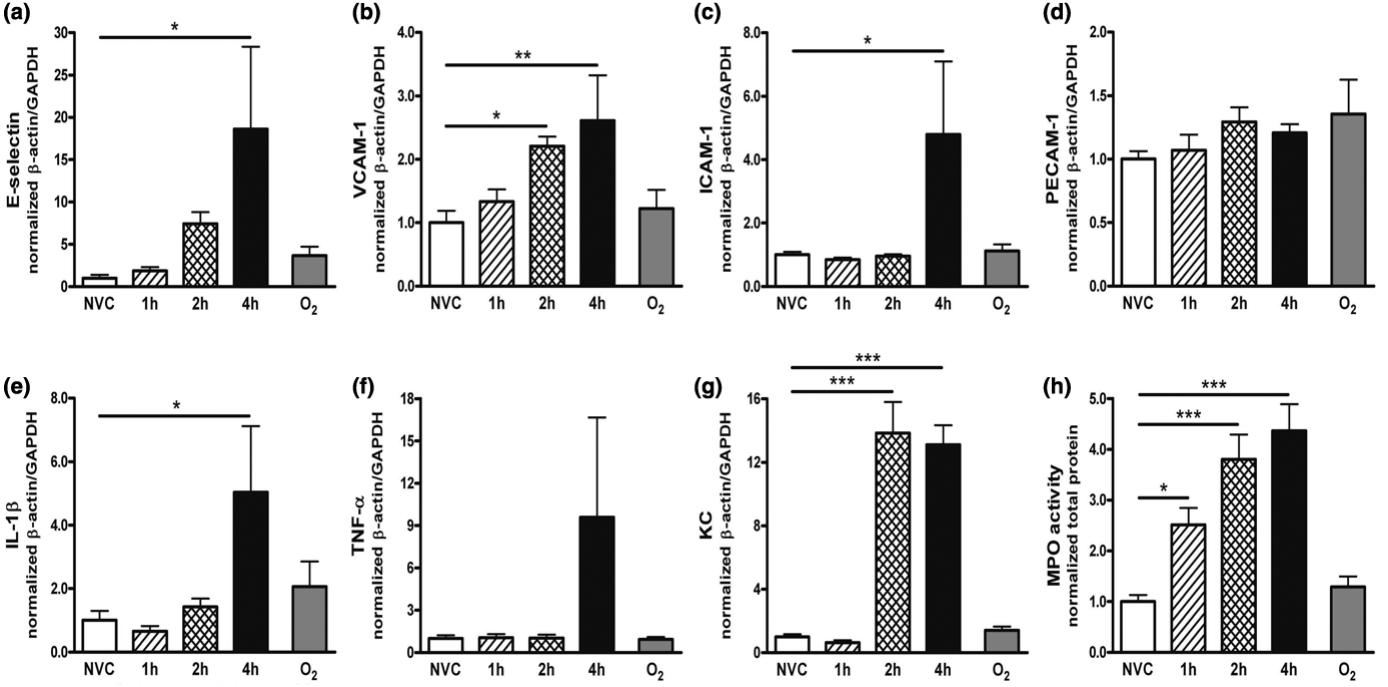
Ventilator-induced endothelial activation and inflammation in hepatic tissue. In total liver homogenates, mRNA expression of the adhesion molecules (a) E-selectin, (b) vascular cell adhesion molecule (VCAM)-1, (c) intercellular adhesion molecule (ICAM)-1 and (d) platelet-endothelial cell adhesion molecule (PECAM)-1 was determined by quantitative real time RT-PCR. In addition, we studied ventilator-induced hepatic inflammation by measuring mRNA expression of the pro-inflammatory cytokines (e) IL-1β and (f) TNF-α and the chemokine (g) keratinocyte-derived chemokine (KC). In total liver homogenates, (h) myeloperoxidase (MPO) activity was determined as a measure of granulocyte infiltration. Data are expressed as mean ± standard error of the mean of four to eight mice for each group (* *P *< 0.05, ** *P *< 0.01, *** *P *< 0.001 vs. non-ventilated controls (NVC)). 1 h = mechanically ventilated for one hour; 2 h = mechanically ventilated for two hours; 4 h = mechanically ventilated for four hours; O_2 _= hyperoxia for four hours; GAPDH = glyceraldehyde 3-phosphate dehydrogenase.

**Figure 4:**
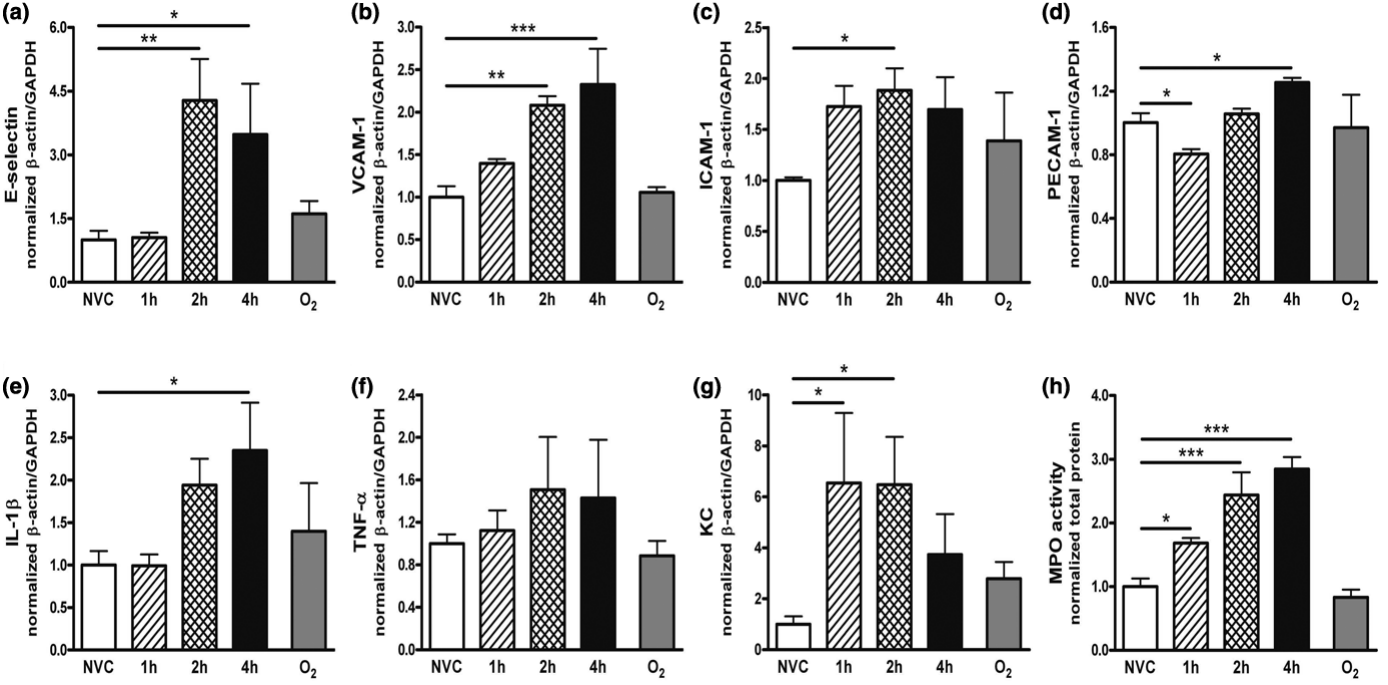
Ventilator-induced endothelial activation and inflammation in renal tissue. In total kidney homogenates, mRNA expression of the adhesion molecules (a) E-selectin, (b) vascular cell adhesion molecule (VCAM)-1, (c) intercellular adhesion molecule (ICAM)-1 and (d) platelet-endothelial cell adhesion molecule (PECAM)-1 was determined by quantitative real time RT-PCR. In addition, we studied ventilator-induced renal inflammation by measuring mRNA expression of the pro-inflammatory cytokines (e) IL-1β and (f) TNF-α and the chemokine (g) keratinocyte-derived chemokine (KC). In total kidney homogenates, (h) myeloperoxidase (MPO) activity was determined as a measure of granulocyte infiltration. Data are expressed as mean ± standard error of the mean of four to eight mice for each group (* *P *< 0.05, ** *P *< 0.01, *** *P *< 0.001 vs. non-ventilated controls (NVC)). 1 h = mechanically ventilated for one hour; 2 h = mechanically ventilated for two hours; 4 h = mechanically ventilated for four hours; O_2 _= hyperoxia for four hours; GAPDH = glyceraldehyde 3-phosphate dehydrogenase.

The effect of MV on endothelial activation in hepatic, renal and cerebral tissue was investigated by analyzing *de novo *synthesis of adhesion molecules. In the liver, higher mRNA expression of E-selectin and ICAM-1 was observed after four hours of MV in comparison with NVC (Figures 3a and 3c). VCAM-1 mRNA was already elevated in hepatic tissue after two hours of MV and further increased after four hours (Figure 3b). No differences were found in PECAM-1 mRNA (Figure 3d). Also in the kidney, we noticed increased mRNA expression of E-selectin, VCAM-1 and ICAM-1 after two and four hours of MV (Figures 4a to 4c). Minimal changes in PECAM-1 mRNA were found in renal tissue of ventilated mice (Figure 4d). In the brain, MV did not induce a significant change in adhesion molecule mRNA expression as compared with NVC (data not shown).

#### Cytokine and chemokine expression

In the liver, IL-1β and TNF-α mRNA expression was enhanced after four hours of MV (Figures 3e and 3f) although the difference in TNF-α mRNA between four hours of MV and NVC did not reach statistical significance (*P *= 0.09). KC mRNA was significantly elevated in hepatic tissue after two hours of MV and further increased after four hours (Figure 3g). In the kidney of ventilated mice, we noticed higher IL-1β mRNA expression at four hours whereas no changes were found in TNF-α mRNA expression (Figures 4e and 4f). Increased KC mRNA was already present after one and two hours of MV (Figure 4g). In the brain, MV did not induce a detectable cytokine or chemokine response (data not shown).

#### Granulocyte recruitment

To determine if enhanced endothelial activation and chemokine expression was accompanied by recruitment of granulocytes to inflamed distal organs, we analyzed MPO activity in hepatic, renal and cerebral tissue. In the liver of ventilated mice, enhanced MPO activity was observed already at one hour and was most pronounced at four hours (Figure 3h). Also in renal tissue, MPO activity was higher after 1 hour of MV and increased further at two and four hours (Figure 4h). In the brain, MPO activity was below detection level in all experimental groups (data not shown).

#### Contribution of hyperoxia

We examined whether the high oxygen levels associated with our MV strategy might contribute to changes in the response of distal organs by exposing spontaneously breathing mice to 100% oxygen levels for four hours. Figures 3 and 4 illustrate that *de novo *synthesis of adhesion molecules, cytokines and chemokines, and MPO activity were comparable in hepatic and renal tissue of hyperoxia-exposed mice and NVC.

## Discussion

To investigate the effects of alveolar stretch on endothelial activation and inflammation in the lung and organs distal to the lung, healthy mice were exposed to a MV strategy that has been described to cause overstretch of pulmonary tissue [24,25]. During four hours of MV, blood gas values remained within the physiological range and pulmonary architecture was preserved suggesting that the cardio-pulmonary integrity was maintained throughout the experiment. Our major finding was that MV induced *de novo *synthesis of various adhesion molecules represented by an elevation of E-selectin and VCAM-1 mRNA in pulmonary tissue and a rise in E-selectin, VCAM-1 and ICAM-1 mRNA in hepatic and renal tissues but not in cerebral tissue. Moreover, we noticed a time-dependent increase in cytokine and chemokine mRNA expression after MV which was accompanied by elevated recruitment of granulocytes. Importantly, this enhanced pro-inflammatory state was found both in the lung and distal organs.

There is convincing evidence that leukocyte-endothelial interactions play a crucial role in the pathogenesis of serious inflammatory diseases related to VILI, such as ALI and ARDS [28,29]. Gando and colleagues observed that soluble levels of P-selectin, E-selectin, ICAM-1 and VCAM-1 were enhanced within 24 hours after the diagnosis of ALI or ARDS [13]. Furthermore, these authors showed a marked increase in these soluble adhesion molecules when subdividing patients into survivors and non-survivors implying that adhesion molecules may have prognostic value for the development and clinical outcome of ALI or ARDS. The present study demonstrates that alveolar stretch imposed by MV induces activation of pulmonary endothelium in healthy mice, as measured by higher mRNA expression of E-selectin and VCAM-1. Our results are supported by *in vitro *models of cyclic strain and shear stress showing increased endothelial expression of adhesion molecules from the selectin family and Ig superfamily [30,31]. Therefore, it appears that ventilator-induced endothelial activation facilitates migration and adhesiveness of activated immune cells to inflamed pulmonary tissue, which in turn may lead to tissue injury. Although MV enhanced the number of granulocytes and expression of pro-inflammatory cytokines or chemokines in the lung, significant changes in pulmonary architecture and oxygenation variables were not observed. In line with this, recent studies demonstrated that MV strategies that do not cause deterioration of pulmonary function *per se *are capable of provoking ventilator-induced lung inflammation [25,32].

To our knowledge, only one other study investigated the effect of MV on expression of cell-bound adhesion molecules. Miyao and colleagues described that ventilation with high tidal volumes enhances P-selectin, VCAM-1 and ICAM-1 expression in pulmonary vasculature of healthy rats [14]. We observed that MV did not only cause up-regulation of adhesion molecules in the lung but also evoked *de novo *synthesis of E-selectin, VCAM-1 and ICAM-1 in organs distal to the lung, such as liver and kidney. Whether a dose response relation exists between the extent of alveolar stretch and effects on distal organs remains to be determined. It has been proposed previously that an elevation of adhesion molecule expression might contribute to tissue injury and ultimately to MOF by facilitating leukocyte activation and migration [22,23]. In line with this notion, we demonstrated that MV augments KC mRNA expression and MPO activity in hepatic and renal tissue. Our data indicate that alveolar stretch due to MV promotes endothelial activation, inflammatory mediator production and the presence of granulocytes in distal organs. Therefore, we propose that MV may play a significant role in the pathogenesis of MOF. Combined with other events, such as an endotoxin challenge, ventilator-induced effects on the lung and distal organs will be exacerbated [33,34] and possibly underlie the high incidence of MOF in critically ill patients ventilated with high pressures and tidal volumes [35].

Studies describing the combined effects of high PaO_2 _levels and MV have revealed that hyperoxia may exacerbate VILI [36,37]. Li and colleagues have shown augmented lung injury in mice exposed to MV with high tidal volumes and hyperoxia compared with animals ventilated with room air [36]. Therefore, we investigated whether the high PaO_2 _levels associated with our MV strategy were contributing to the observed changes in expression of adhesion molecules, cytokines and chemokines, and recruitment of granulocytes. In our study, hyperoxia as such did not lead to pulmonary endothelial activation and inflammation. Furthermore, we noticed that the high PaO_2 _levels did not induce an augmented immune response in organs distal to the lung. Although we cannot exclude that hyperoxia is aggravating the stretch-induced inflammatory response in pulmonary tissue, we consider that effects of high PaO_2 _levels on the inflammatory state of the liver and kidney will not be the primary cause of distal organ activation. As the reference group in our study (NVC) could not be sedated for the same period as ventilated animals, we cannot exclude that anesthesia affects endothelial activation and inflammation by itself. However, we have previously shown that MV with injurious settings (PIP 32/PEEP 6) increased pulmonary macrophage inflammatory protein-2 expression and reduced splenocyte natural killer cell activity whereas MV with protective settings (PIP 14/PEEP 6) did not have these effects on inflammation [24]. Since the same type of anesthesia was applied in the two abovementioned groups, we propose that anesthesia as such does not induce the inflammatory response.

Taken together, we demonstrated that MV induces endothelial activation and inflammation in the lung but also in the liver and kidney. It remains to be determined which factors lead to the onset of this inflammatory response in distal organs. Haitsma and colleagues and Tutor and colleagues have shown that ventilator-induced permeability of the alveolar-capillary barrier causes release of inflammatory mediators into the systemic circulation [38,39]. In the present study, alveolar stretch imposed by MV enhanced mRNA expression of adhesion molecules and cytokines or chemokines in hepatic and renal tissue, thus inducing *de novo *synthesis of these mediators in organs distal to the lung. Furthermore, we observed granulocyte recruitment to the liver and kidney, and KC mRNA expression in the kidney already after one hour of MV. These results indicate that ventilator-induced changes of the immune response may occur simultaneously in the lung, liver and kidney, and imply that release of inflammatory mediators into the circulation is probably not the only cause of augmented endothelial activation or inflammation in distal organs. We cannot exclude, however, that cytokines in the systemic circulation induce *de novo *synthesis of adhesion molecules and cytokines or chemokines in distal organs.

It has been hypothesized that the physical stress of MV activates the sympathetic nervous system [19]. In this regard, Elenkov and colleagues and Straub and colleagues have proposed that stimulation of sympathetic nerve terminals evokes an inflammatory response in peripheral organs [40,41]. Catecholamines activate transcription factors such as nuclear factor kappa B in macrophages thereby promoting IL-1, TNF and IL-8 production, which in turn might result in an acute phase response in the liver, possibly via α-adrenergic activation [40,42,43]. Therefore, the systemic endothelial activation and inflammation caused by ventilator-induced alveolar stretch may be explained by activation of sympathetic nerve terminals in organs distal to the lung. If so, blockade of adrenergic receptor function will give further insight into the mechanism of distal organ inflammation. Future studies should also aim to develop intervention strategies to prevent simultaneous endothelial activation or inflammation in the lung and distal organs during MV. Such intervention strategies may not only improve the efficacy of MV but could also contribute to preventing MOF.

## Conclusions

We have shown that alveolar stretch imposed by four hours of MV did not only provoke *de novo *synthesis of adhesion molecules and recruitment of granulocytes in the lung but also in organs distal to the lung such as liver and kidney, although not the brain. Our results demonstrate that ventilator-induced endothelial activation and inflammation in both the lung and distal organs may be crucial factors in the pathogenesis of MOF.

## Key messages

• Alveolar stretch imposed by MV induces *de novo *synthesis of adhesion molecules, cytokines and chemokines in the lung.

• Alveolar stretch imposed by MV induces *de novo *synthesis of adhesion molecules, cytokines and chemokines in organs distal to the lung, such as liver and kidney.

• Ventilator-induced endothelial activation and inflammation in both the lung and distal organs may be crucial factors in the pathogenesis of MOF.

## Abbreviations

ALI: acute lung injury; ARDS: acute respiratory distress syndrome; BE: base excess; BSA: bovine serum albumin; ELISA: enzyme-linked immunosorbent assay; FiO_2_: fractional inspired oxygen concentration; H&E: hematoxylin and eosin; ICAM: intercellular adhesion molecule; Ig: Immunoglobulin; IL: interleukin; KC: keratinocyte-derived chemokine; MOF: multiple-organ failure; MPO: myeloperoxidase; MV: mechanical ventilation; NVC: non-ventilated controls; PaCO_2_: partial pressure of arterial carbon dioxide; PaO_2_: partial pressure of arterial oxygen; PBS: phosphate-buffered saline; PECAM: platelet-endothelial cell adhesion molecule; PEEP: positive end expiratory pressure; PIP: peak inspiratory pressure; RT-PCR: reverse transcriptase polymerase chain reaction; TNF: tumor necrosis factor; VCAM: vascular cell adhesion molecule; VILI: ventilator-induced lung injury.

## Competing interests

The authors declare that they have no competing interests.

## Authors' contributions

MAH performed the experimental work, interpreted the results and drafted the manuscript. MPH and PS performed the experimental work and were responsible for critical review of the manuscript. BL participated in study design and was responsible for critical review of the manuscript. CH, NJ, AV and PC supervised the study, were involved in interpreting the results and correcting the manuscript. All authors have read and have approved the final version of the manuscript.
